# Herpes Zoster Reactivation Following COVID-19 and the Risk of Renal, Infectious, and Autoimmune Complications: A Global Propensity-Matched Cohort Study

**DOI:** 10.3390/biomedicines13071628

**Published:** 2025-07-02

**Authors:** Ming-Hung Chien, Joshua Wang, Kuo-Cheng Lu, Chien-Lin Lu

**Affiliations:** 1 Division of Nephrology, Department of Internal Medicine, Fu Jen Catholic University Hospital, Fu Jen Catholic University, New Taipei City 24352, Taiwan; a02786@mail.fjuh.fju.edu.tw; 2 Department of Research, Taipei Tzu Chi Hospital, Buddhist Tzu Chi Medical Foundation, New Taipei City 23142, Taiwan; 3 School of Biomedical Sciences, Queensland University of Technology, Brisbane, QLD 4001, Australia; 4 Division of Nephrology, Department of Medicine, Taipei Tzu Chi Hospital, Buddhist Tzu Chi Medical Foundation, New Taipei City 23142, Taiwan; 5 School of Medicine, College of Medicine, Fu Jen Catholic University, New Taipei City 24205, Taiwan

**Keywords:** autoimmune disease, COVID-19, herpes zoster, kidney injury, rheumatoid arthritis, sepsis

## Abstract

**Background:** Herpes zoster (HZ), resulting from the reactivation of latent varicella-zoster virus, has been increasingly observed in individuals following COVID-19. Given the shared immunological disturbances between the two conditions, this study aimed to investigate whether HZ following COVID-19 is associated with an elevated risk of renal, infectious, and autoimmune complications. **Methods:** This retrospective cohort study utilized data from the TriNetX global federated health network, encompassing over 9 million adults diagnosed with COVID-19 between January 2020 and January 2022. Patients who developed HZ within one year following COVID-19 diagnosis were compared to 1:1 propensity score-matched controls without HZ. Time-to-event analyses over a three-year follow-up period were conducted to estimate the risks of major adverse kidney events (MAKE; defined as acute kidney injury, dialysis dependence, or severely reduced kidney function with eGFR <30 mL/min/1.73 m^2^), sepsis, systemic lupus erythematosus (SLE), and rheumatoid arthritis (RA), using Kaplan–Meier survival curves and Cox proportional hazards models. **Results:** HZ following COVID-19 was significantly associated with increased risks of all four outcomes: MAKE (HR 1.940, 95% CI: 1.866–2.017), sepsis (HR 2.362, 95% CI: 2.250–2.479), SLE (HR 2.667, 95% CI: 2.254–3.156), and RA (HR 2.484, 95% CI: 2.267–2.730). Subgroup analyses identified older age, diabetes, impaired renal function, and elevated inflammatory markers as key risk-enhancing factors. **Conclusions:** HZ following COVID-19 may serve as a clinical indicator of systemic immune dysregulation and is independently associated with increased long-term risks of renal, infectious, and autoimmune sequelae. Enhanced monitoring of this high-risk population is warranted.

## 1. Introduction

The coronavirus disease 2019 (COVID-19) pandemic has exerted profound and sustained impacts on global health, with millions of survivors experiencing long-term complications beyond the acute phase [1]. Among these, concerns about persistent immune dysregulation have led to increased attention toward viral reactivations and their systemic consequences [2,3]. Herpes zoster (HZ), caused by reactivation of latent varicella-zoster virus (VZV), has emerged as a potential immunologic sequela in the post-COVID setting [4,5]. Multiple case reports and cohort studies suggest a temporal relationship between SARS-CoV-2 infection and subsequent HZ episodes, possibly mediated by T-cell exhaustion, immunosenescence, and disruption of antiviral immunity [4,6,7]. Population-level evidence has also begun to emerge. A multinational study from South Korea, Japan, and the UK reported an increase in incident allergic and viral conditions, including HZ, following COVID-19, particularly among children, implying broad immune alterations after infection [8].

Beyond its dermatologic manifestations, HZ has been associated with systemic complications such as neurologic disorders, cardiovascular events, and kidney injury [9,10,11]. These events may result from both direct viral effects and an exaggerated inflammatory response involving endothelial injury and cytokine imbalance. Notably, HZ and COVID-19 share overlapping immunopathological features—including lymphopenia, impaired interferon signaling, and heightened inflammatory cytokines—which may act synergistically to amplify immune dysfunction [12,13,14].

Reactivation of latent herpesviruses (e.g., cytomegalovirus, Epstein–Barr virus, herpes simplex virus (HSV)) has been frequently observed in critically ill or immunocompromised individuals and is often associated with adverse outcomes such as organ failure, prolonged hospitalization, and mortality [15,16,17,18]. These episodes likely reflect systemic immune vulnerability under physiological stress. Thus, HZ occurring after COVID-19 may represent more than a dermatologic complication—it may serve as a sentinel marker of broader immune dysregulation and systemic risk.

However, current evidence remains limited to small-scale or single-center studies, and the long-term prognostic significance of post-COVID HZ reactivation has not been adequately examined in large, population-based datasets. To address this gap, we leveraged a global real-world database to assess the clinical trajectory of patients who developed HZ after COVID-19. Specifically, we aimed to determine whether post-COVID HZ reactivation is associated with increased risks of renal, infectious, or autoimmune complications—thereby evaluating its potential role as a clinical signal of underlying immune imbalance.

## 2. Methods

### 2.1. Study Framework and Data Source

This retrospective, multicenter cohort analysis was conducted using the TriNetX Analytics Platform, a global federated research network that analyzes anonymized electronic health records (EHRs) from participating healthcare institutions. The study accessed data from the Global Collaborative Network within TriNetX, which includes over 140 healthcare organizations worldwide, with approximately 90% of clinical facts recorded in the United States. The database encompasses structured clinical data such as diagnoses, procedures, laboratory results, prescriptions, and vital status. All analyses adhered to international privacy standards including HIPAA and GDPR. The study was approved by the Institutional Review Board of Taipei Tzu Chi Hospital (Approval No. 14-IRB043), which waived the requirement for informed consent due to the use of de-identified retrospective data. This report follows the STROBE (Strengthening the Reporting of Observational Studies in Epidemiology) guidelines for observational research.

### 2.2. Cohort Assembly and Exposure Definition

**Figure 1:**
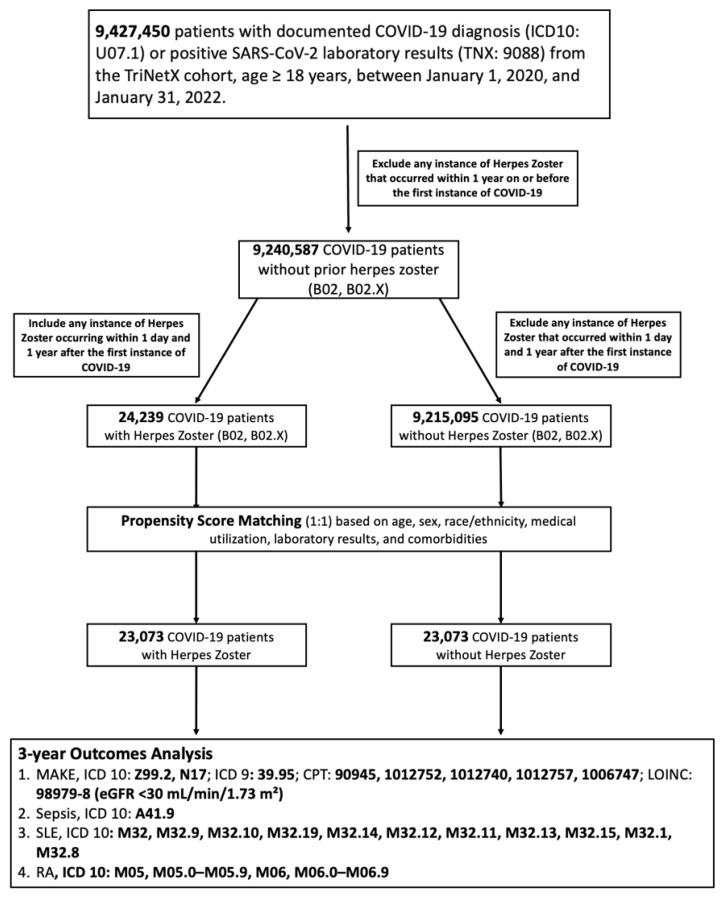
Cohort selection flowchart and outcome definitions. Abbreviations: COVID-19, coronavirus disease 2019; CPT, current procedural terminology; eGFR, estimated glomerular filtration rate; HZ, herpes zoster; ICD, International Classification of Diseases; LOINC, Logical Observation Identifiers Names and Codes; MAKE, major adverse kidney events; PSM, propensity score matching; RA, rheumatoid arthritis; SLE, systemic lupus erythematosus.

The study population consisted of adult individuals (≥18 years old) with a documented diagnosis of COVID-19 between 1 January 2020 and 31 January 2022. COVID-19 cases were identified based on either a positive SARS-CoV-2 nucleic acid test (TNX:9088) or International Classification of Diseases, 10th Revision, Clinical Modification (ICD-10-CM) code U07.1. Patients were stratified into two exposure groups: those who developed HZ within one year following their first recorded COVID-19 diagnosis or positive test result, and those without any HZ diagnosis throughout the observation window. HZ was defined using ICD-10-CM codes B02, including all subcategories (B02.1–B02.9, B02.X). Individuals with documented HZ prior to their first recorded COVID-19 diagnosis or positive test result were excluded to minimize reverse causality. After initial screening, 24,239 patients with HZ and over 9.2 million without HZ were identified prior to matching. The cohort selection process is illustrated in Figure 1.

### 2.3. Index Date and Outcome Observation Period

For both cohorts, the index date was defined as the date of first confirmed COVID-19 diagnosis or positive SARS-CoV-2 test. The outcome evaluation window extended from day 1 after the index event to a maximum of 1095 days (3 years), or until patient death or last clinical encounter, whichever occurred first. Any outcome events that occurred prior to the index or within the first day of follow-up were excluded from all time-to-event and risk calculations.

### 2.4. Propensity Score Matching

To address baseline imbalances in patient characteristics between cohorts and minimize confounding, 1:1 propensity score matching (PSM) was conducted using a greedy nearest-neighbor algorithm without replacement. Covariates incorporated into the matching model included patient demographics, comorbidities, medication usage, and relevant laboratory values. Matching was implemented across both cohorts to generate demographically balanced groups (*n* = 5208 per arm). Covariate balance was assessed using standardized mean differences (SMDs), with values <0.1 considered indicative of adequate matching. Post-matching assessment confirmed that most covariates were well balanced (Table 1). Minor residual imbalances were noted for serum ferritin and lactate levels, with SMDs slightly exceeding 0.1, potentially reflecting differences in iron metabolism or systemic stress not fully addressed by PSM.

Due to technical constraints within the TriNetX platform when handling large datasets exceeding 9 million records, the matching was conducted within a restricted age range of 50–60 years to ensure computational feasibility and statistical rigor. This subgroup was selected as a clinically relevant and demographically representative sample of the overall adult cohort. To validate the robustness and generalizability of findings derived from the age-restricted matched sample, parallel analyses of three-year outcomes—including major adverse kidney events (MAKE), sepsis, systemic lupus erythematosus (SLE), and rheumatoid arthritis (RA)—were independently performed in both the full unmatched cohort and the age-restricted matched population. The comparable patterns of risk estimates and outcome distributions across these two analyses (Supplementary Table S1) support the external validity of the matched cohort findings.

### 2.5. Study Outcomes

The primary outcomes of interest included MAKE, sepsis, SLE, and RA. These outcomes were defined using standardized ICD-10-CM and Current Procedural Terminology (CPT) codes available within the TriNetX platform. To ensure accurate temporal associations between exposure and outcomes, patients with any documentation of the respective outcome prior to or on the index date were excluded from the relevant analysis.

MAKE was operationalized as a composite indicator of serious kidney-related outcomes, encompassing both diagnostic and procedural codes. Specifically, this included dialysis dependence (ICD-10-CM: Z99.2), acute kidney injury (AKI) (ICD-10-CM: N17), and dialysis procedures identified via CPT (90945, 1012752, 1012740, 1012757 and 1006747) and ICD-9-CM (39.95) codes. Additionally, the MAKE definition incorporated the most recent estimated glomerular filtration rate (eGFR) calculated using the Chronic Kidney Disease Epidemiology Collaboration (CKD-EPI) equation, with values below 30 mL/min/1.73 m^2^ identified by the Logical Observation Identifiers Names and Codes (LOINC) system (LOINC: 98979-8). Sepsis was identified using the ICD-10-CM code A41.9, representing sepsis caused by an unspecified organism. Patients with prior sepsis diagnoses were excluded from the analysis to preserve the temporal relationship with COVID-19 and HZ onset. SLE was defined using a comprehensive range of ICD-10-CM codes from the M32 series, capturing both general and organ-specific manifestations such as pericarditis (M32.12), endocarditis (M32.11), glomerulonephritis (M32.14), pulmonary involvement (M32.13), and tubulointerstitial nephropathy (M32.15). RA was defined using ICD-10-CM codes in the M05 and M06 categories, including seropositive and seronegative variants, and systemic complications like Felty’s syndrome, rheumatoid vasculitis, and rheumatoid-related pulmonary or cardiac disease.

### 2.6. Statistical Analysis

Following matching, outcome comparisons were conducted using two analytic approaches. Cumulative incidence rates, absolute risk differences, relative ratios (RRs), and odd ratios (ORs) were calculated using the TriNetX Risk Analysis module, with exclusion of patients with prior outcome history. Time-to-event outcomes were assessed using Kaplan–Meier survival curves and compared via log-rank tests. Cox proportional hazards models were employed to estimate hazard ratios (HRs) and 95% CIs for each outcome. All statistical tests were two-tailed, with significance defined as *p* < 0.05. Analyses were conducted natively within the TriNetX platform environment.

### 2.7. Sensitivity Analyses

To assess the robustness and generalizability of our primary findings, two sensitivity analyses were conducted: First, we tested whether the associations between HZ and adverse outcomes were consistent across different age strata. A parallel survival analysis was conducted comparing the full adult population (aged ≥ 18 years) with a restricted subgroup aged 50–60 years. For both cohorts, HRs were derived from time-to-event analyses of MAKE, sepsis, SLE, and RA. The purpose of this age-restricted analysis was to evaluate whether the observed associations persisted within a narrower and clinically relevant age band, thus reinforcing the external validity of the main results (Supplementary Table S1).

Second, we evaluated the 3-year risks of MAKE, sepsis, SLE, and RA using Kaplan–Meier survival analyses both before and after PSM. This comparison allowed us to determine whether the associations between HZ and long-term complications remained robust despite differences in baseline covariates. Consistent effect sizes across matched and unmatched cohorts would support that the observed risks are not solely attributable to confounding (Supplementary Table S2).

## 3. Results

### 3.1. Major Adverse Kidney Events (MAKE)

**Figure 2:**
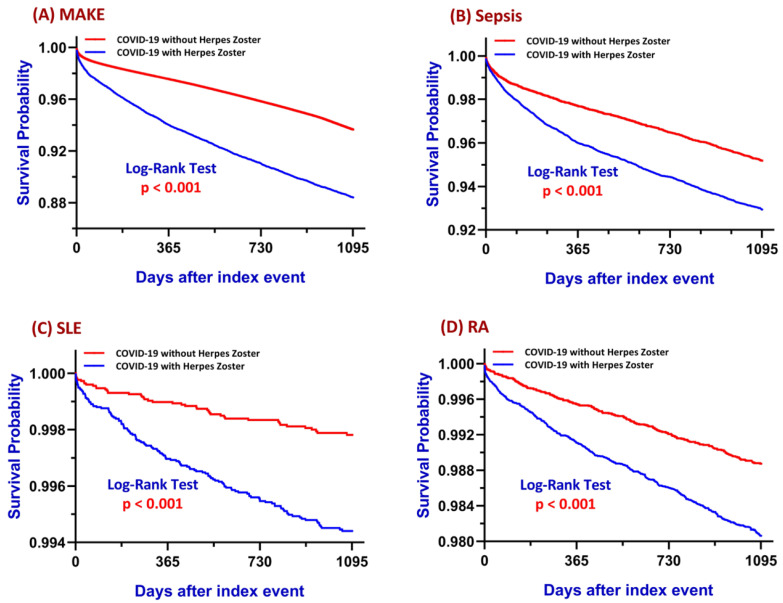
Kaplan–Meier survival curves comparing COVID-19 patients with and without herpes zoster (HZ) infection for (**A**) major adverse kidney events (MAKE), (**B**) sepsis, (**C**) systemic lupus erythematosus (SLE), and (**D**) rheumatoid arthritis (RA) over a three-year follow-up period. The blue line represents patients with HZ, and the red line represents those without HZ. The log-rank test was used to compare survival distributions between groups, with *p*-values provided in each panel. Abbreviations: COVID-19, coronavirus disease 2019; HZ, herpes zoster.

In the matched cohort, patients diagnosed with HZ within one year following COVID-19 exhibited a significantly higher incidence of MAKE compared to their non-HZ counterparts (10.6% vs. 4.1%). The absolute risk difference was 6.5% (95% CI: 6.1–6.9%), corresponding to a RR of 2.590 and an OR of 2.778 (95% CI: 2.667–2.895; *p* < 0.001). Kaplan–Meier survival analysis demonstrated a significantly lower MAKE-free survival probability in the HZ group, with a log-rank *p*-value < 0.001 and a HR of 1.940 (95% CI: 1.866–2.017), suggesting a nearly twofold increase in time-to-event risk of MAKE associated with post-COVID HZ (Figure 2A).

**Figure 3:**
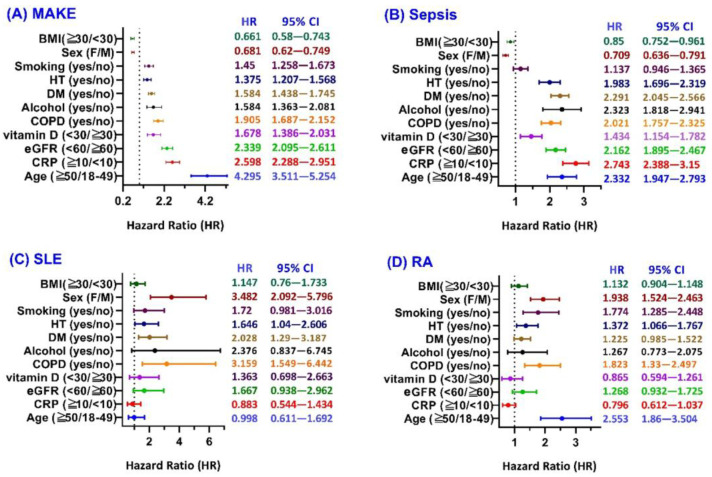
Subgroup analyses of risk factors associated with post-COVID herpes zoster–related outcomes. Hazard ratios (HRs) and 95% confidence intervals (CIs) for key covariates associated with four outcomes: (**A**) major adverse kidney events (MAKE), (**B**) sepsis, (**C**) systemic lupus erythematosus (SLE), and (**D**) rheumatoid arthritis (RA). Abbreviations: BMI, body mass index; COPD, chronic obstructive pulmonary disease; CRP, C-reactive protein; DM, diabetes mellitus; eGFR, estimated glomerular filtration rate; HT, hypertension.

Subgroup analysis (Figure 3A) revealed that several factors were significantly associated with an increased risk of MAKE following post-COVID HZ. These included smoking, hypertension, diabetes mellitus, alcohol use, chronic obstructive pulmonary disease (COPD), vitamin D deficiency, reduced renal function (eGFR < 60), elevated C-reactive protein (CRP ≥ 10), and older age (≥50 years). Conversely, female sex and higher body mass index (BMI ≥ 30) were significantly associated with a lower risk of MAKE. These findings indicate that post-COVID HZ is significantly associated with an increased risk of MAKE.

### 3.2. Sepsis

The incidence of sepsis was significantly higher among patients with HZ following COVID-19 compared to those without HZ (6.5% vs. 3.6%). This corresponded to an absolute risk difference of 2.8% (95% CI: 2.5–3.2%), with a RR of 1.781 and OR of 1.835 (95% CI: 1.693–1.989; *p* < 0.001). Kaplan–Meier analysis indicated lower sepsis-free survival in the HZ group, confirmed by the log-rank test (*p* < 0.001), and the HR was 2.362 (95% CI: 2.250–2.479), suggesting a sustained heightened risk of sepsis following HZ (Figure 2B).

As shown in Figure 3B, the risk of sepsis following post-COVID HZ was significantly elevated among individuals with hypertension, diabetes mellitus, alcohol use, COPD, vitamin D deficiency, impaired renal function, elevated CRP, and age ≥ 50 years. Female sex and BMI ≥ 30 were again associated with a lower risk, while smoking did not show a significant effect in this outcome. These results suggest that post-COVID HZ is significantly associated with an increased risk of sepsis.

### 3.3. Systemic Lupus Erythematosus

Among matched cohorts, patients diagnosed with HZ within one year following COVID-19 exhibited a higher incidence of SLE than those without HZ (0.5% vs. 0.2%). This yielded an absolute risk difference of 0.3% (95% CI: 0.2–0.4%), a RR of 3.080, and an OR of 3.091 (95% CI: 2.214–4.314; *p* < 0.001). Kaplan–Meier survival analysis confirmed significantly lower SLE-free survival in the HZ group (log-rank *p* < 0.001), with a corresponding HR of 2.667 (95% CI: 2.254–3.156), indicating a sustained and significantly increased risk of incident SLE over time (Figure 2C).

Subgroup analysis for SLE (Figure 3C) demonstrated a significantly increased risk among females, patients with hypertension, diabetes, and COPD. However, other subgroups—including BMI, smoking status, alcohol use, vitamin D status, renal function, CRP level, and age—did not show statistically significant associations with SLE risk. These findings indicate that post-COVID HZ is significantly associated with an elevated risk of developing SLE.

### 3.4. Rheumatoid Arthritis

In the matched cohort, the incidence of RA was significantly higher among patients who developed HZ after COVID-19 compared to those who did not (1.7% vs. 0.8%). The absolute risk difference was 0.9% (95% CI: 0.7–1.1%), with a RR of 2.104 and an OR of 2.124 (95% CI: 1.809–2.494; *p* < 0.001). Kaplan–Meier analysis confirmed significantly lower RA-free survival in the HZ group (log-rank *p* < 0.001), with a HR of 2.488 (95% CI: 2.267–2.730), indicating a persistent and elevated risk of RA development in this population (Figure 2D).

In the RA subgroup analysis (Figure 3D), significant risk factors included female sex, smoking, hypertension, and COPD. Other subgroups—BMI, diabetes, alcohol use, vitamin D deficiency, reduced eGFR, elevated CRP, and age—did not demonstrate statistically significant associations with RA development. These results suggest that post-COVID HZ is significantly associated with an increased risk of developing RA.

### 3.5. Timing and Prevalence of COVID-19 Vaccination Relative to HZ

Among patients diagnosed with HZ following COVID-19, we explored the timing of COVID-19 vaccination relative to HZ onset. As shown in Supplementary Table S5 and Supplementary Figure S1, vaccination prevalence exhibited a bell-shaped distribution across the 36-month window. The percentage of vaccinated individuals increased progressively from 1.35% (15–18 months prior to HZ) to a peak of 5.96% during the 0–3 months after HZ, and then declined symmetrically to 2.31% in the 15–18 months post-HZ period. This temporal pattern suggests clustering of COVID-19 vaccination within 3 months before or after HZ reactivation.

### 3.6. Sensitivity Analysis

To assess the robustness of the findings, two sets of sensitivity analyses were conducted (Supplementary Tables S1 and S2). The first analysis compared the risks of MAKE, sepsis, SLE, and RA before and after PSM. HRs remained consistently elevated and statistically significant (*p* < 0.0001) across all outcomes, demonstrating that the associations between HZ and adverse events were robust to baseline covariate adjustment.

The second analysis focused on an age-restricted subgroup (50–60 years) versus the full cohort. HRs in the age-restricted group were slightly lower in this age group but outcome risks remained significantly increased in the HZ cohort for all outcomes, confirming that the associations between HZ and MAKE, sepsis, SLE, and RA persisted even in middle-aged patients (Supplementary Table S2).

To address surveillance bias, an additional sensitivity analysis was performed using an expanded matching model that incorporated ICD-10 Z-codes reflecting healthcare utilization (e.g., general checkups and follow-ups). As shown in Supplementary Tables S3 and S4, Z-code variables were well balanced after matching (all SMDs < 0.01), and HRs for all outcomes remained statistically significant despite attenuation (e.g., MAKE HR reduced from 1.940 to 1.587), suggesting that increased medical contact alone does not account for the observed associations.

## 4. Discussion

This global cohort study demonstrates that a diagnosis of HZ following COVID-19 is independently associated with significantly increased risks of long-term renal, infectious, and autoimmune complications. Post-matching balance was achieved for nearly all covariates, with only minor residual imbalances in ferritin and lactate (Table 1). Elevated ferritin, an acute-phase reactant, may indicate underlying low-grade inflammation or immune activation and has been linked to poor renal outcomes, greater sepsis risk, and increased autoimmune activity [19,20]. Similarly, elevated lactate may reflect mitochondrial dysfunction or tissue hypoperfusion—both associated with adverse outcomes in sepsis, kidney injury, and systemic inflammation [21,22]. Although these variables were not adjusted post hoc due to small effect sizes, their biological relevance suggests that unresolved inflammation and metabolic stress may partially explain the observed associations in the HZ following COVID-19 cohort. Subgroup analysis further confirmed that vitamin D deficiency was significantly associated with increased risk of MAKE and sepsis (Figure 3A,B), indicating that nutritional immune modulation may further influence post-infectious outcomes.

When comparing the observed outcomes, MAKE and sepsis presented with the highest absolute risks (10.7% and 6.5%, respectively), whereas autoimmune outcomes such as RA (1.7%) and SLE (0.5%) were less frequent but still showed significantly elevated relative risks. These distinctions may reflect differences in the latency, pathophysiology, and immune mechanisms underlying each condition. Acute outcomes like MAKE and sepsis are typically driven by systemic inflammation, immune dysregulation, and organ perfusion deficits, whereas SLE and RA involve chronic autoimmune activation and delayed onset. The greater incidence of sepsis versus SLE suggests a more immediate post-infectious vulnerability to infectious complications, while the comparatively higher RA risk versus SLE (1.7% vs. 0.5%) may point to differing thresholds for autoimmune activation across disease types. This suggests that post-COVID-19 HZ is associated with higher relative risk but differing absolute burdens across renal, infectious, and autoimmune outcomes.

Subgroup analyses revealed that age ≥50 years was a consistent risk factor for MAKE, sepsis, and RA, but not SLE. This suggests that advancing age may exacerbate immune dysregulation and promote the development of chronic inflammatory diseases, with the exception of SLE, which predominantly affects younger women. Regarding sex and BMI, divergent effects were observed across outcomes. Female sex and higher BMI conferred significant protection against MAKE and sepsis; however, only female sex—not BMI—was associated with increased risk of autoimmune outcomes. The protective role of female sex in acute outcomes may reflect stronger innate and adaptive immune responses in women, facilitating more efficient pathogen clearance and reduced end-organ damage [23]. Although estrogen has been shown to have anti-inflammatory properties [24], the current study did not measure estrogen levels. Therefore, while sex differences in immune function may contribute to outcome variation, attributing the observed risks solely to estrogen would be speculative and is not directly supported by our dataset. In contrast, women exhibited significantly higher risks of SLE and RA, in line with the established female predominance of these conditions. This increased susceptibility may be attributed to X-linked immune gene expression and estrogen-driven B cell hyperactivity, which promote autoantibody production and immune dysregulation [25,26]. Viral infections such as COVID-19 and HZ may further disrupt immune balance, thereby unmasking or accelerating autoimmune disease onset in susceptible women [27,28].

Similarly, while elevated BMI is generally a risk factor for chronic disease, it appeared protective in acute outcomes such as sepsis and AKI. This supports the “obesity paradox”, in which greater energy reserves and better nutritional status may help buffer acute metabolic stress [29,30]. However, this protection did not extend to SLE or RA, where adiposity is known to drive chronic low-grade inflammation via cytokines such as IL-6 and TNF-α, which are also central to autoimmune pathogenesis [31].

The strong association between HZ following COVID-19 and MAKE may be explained by several proposed mechanisms. Reports of HZ-associated AKI in CKD or transplant patients suggest direct viral or immune-mediated nephrotoxic effects, even during the prodromal phase [32]. Laboratory findings such as hyponatremia and metabolic acidosis further imply systemic inflammation and tubular injury [33]. Acyclovir-induced nephrotoxicity is another concern, especially with intravenous administration [34]. Crystalluria and acute tubular necrosis have been commonly reported, particularly in patients with inadequate hydration [35]. Although our dataset did not include treatment information, these risks underscore the importance of antiviral selection and adequate hydration in managing post-HZ renal injury. In rare cases, HZ-associated rhabdomyolysis has caused pigment nephropathy and irreversible damage, highlighting the variety of renal conditions observed in the context of viral reactivation [36,37].

The twofold increase in sepsis risk observed in patients with HZ following COVID-19 is also noteworthy. Reactivation of latent herpesviruses such as VZV and HSV is known to mimic or trigger sepsis, particularly in patients with CKD or compromised immunity [38,39]. Critically ill patients have developed herpesvirus viremia even in the absence of immunosuppression, and such events are associated with worse ICU outcomes [40]. Immune paralysis after COVID-19 or sepsis—marked by T-cell exhaustion and cytokine suppression—may explain why HZ may serve as a marker or correlate of systemic immune dysfunction in this context [41,42]. Cases of fulminant viral sepsis due to HSV or VZV, even in immunocompetent hosts, support this mechanism, particularly when the liver or central nervous system is involved [43]. Compared to MAKE, sepsis occurred less frequently but demonstrated a higher hazard ratio (HR 2.362 vs. HR 1.904), suggesting a more sharply elevated relative risk for infectious complications in this population.

SLE and RA are classic autoimmune conditions characterized by chronic inflammation and immune dysregulation [44]. Emerging data suggest that viral triggers—including SARS-CoV-2—may play a role in initiating these diseases. Several case reports and reviews have described new-onset SLE following COVID-19, typically occurring within weeks and involving renal dysfunction, lymphopenia, and autoantibody positivity [45,46]. Mechanistic studies implicate enhanced type I interferon signaling, B cell hyperactivation, and cytokine imbalance—central mechanisms in lupus pathogenesis [47,48]. One review identified ten post-COVID SLE cases, most of which required immunosuppressive therapy and achieved partial remission [45].

Our findings further suggest that HZ reactivation in the post-COVID setting may act as a co-factor or marker for immune imbalance preceding SLE. The SLE incidence was significantly higher in the HZ group (0.5%) compared to controls (0.2%), corresponding to a hazard ratio of 2.667. However, the absolute risk difference was modest (0.3%), and should be interpreted with caution given the low overall incidence. These results support a model in which viral reactivation may contribute to or coincide with activation of autoreactive immune cascades, particularly in predisposed individuals. Prior studies have noted that HZ episodes frequently occur around the time of SLE diagnosis—even without immunosuppressive use—suggesting viral priming may break immune tolerance [49]. Autoantibody patterns such as anti-Sjögren’s-syndrome-related antigen A antibody (anti-Ro) and anti-ribonucleoprotein (anti-RNP), associated with both SLE and heightened HZ risk, further indicate a shared immunologic pathway involving viral antigens and self-reactivity [50].

RA similarly showed increased incidence in the HZ cohort (1.7% vs. 0.8%; HR 2.488), yielding an absolute difference of 0.9%. This difference, while numerically greater than that observed for SLE, still represents a relatively low incidence in the context of population risk assessment. Viral infections have been proposed to enhance inflammatory signaling through IL-6, TNF-α, and IFNs, all key players in joint inflammation and autoimmune progression [51,52]. In our subgroup analyses, significantly increased RA risk following HZ was observed in females and those with smoking history, hypertension, COPD, or aged ≥50 years. In contrast, the SLE subgroup analyses showed stronger associations in females and those with hypertension, diabetes mellitus, or COPD. These results underscore that host factors—such as sex, comorbidities, and age—may differentially influence immune vulnerability to autoimmune sequelae following viral reactivation.

In summary, the spectrum of risks associated with post-COVID-19 HZ varies in magnitude and pathophysiologic basis across MAKE, sepsis, SLE, and RA. While MAKE and sepsis represent more immediate, acute complications driven by inflammatory and metabolic derangements, SLE and RA reflect downstream autoimmune dysregulation likely exacerbated by latent viral reactivation. These findings highlight the multifaceted nature of HZ-related complications in COVID-19 survivors and emphasize the importance of vigilant long-term monitoring across both infectious and immune-mediated domains. This suggests that post-COVID-19 HZ is associated with both short- and long-term health burdens spanning renal, infectious, and autoimmune outcomes, requiring integrative clinical awareness.

Interestingly, vitamin D deficiency demonstrated divergent, though non-significant, associations with SLE and RA in our stratified analyses. VDD was associated with a non-significant increase in SLE risk (HR 1.363, 95% CI: 0.698–2.663) but a non-significant decrease in RA risk (HR 0.865, 95% CI: 0.594–1.261). These results should be interpreted cautiously due to wide confidence intervals and limited statistical power. The divergent trends may reflect distinct immunoregulatory mechanisms. For instance, SLE pathogenesis involves B cell hyperactivation and type I interferon signaling, both of which may be exacerbated by VDD-related immune dysregulation [53,54,55]. In contrast, vitamin D’s role in modulating Th17/Treg balance in RA appears more complex and may be influenced by disease stage, baseline inflammation, or concurrent use of immunomodulatory therapies [56,57,58]. These findings are hypothesis-generating rather than definitive, and further mechanistic or prospective studies are needed to elucidate the immunologic effects of vitamin D in distinct autoimmune contexts. Given the observational nature of our study, all findings should be interpreted as associations rather than causal relationships.

While our study was not designed to investigate vaccine-induced HZ, we examined the temporal relationship between COVID-19 vaccination and HZ reactivation. Supplementary Figure S1 reveals that vaccination rates peaked in the 0–3 months before and after HZ diagnosis, consistent with prior case reports linking mRNA-based vaccines to VZV reactivation [59,60]. However, due to limitations in the TriNetX platform, we could not ascertain the specific vaccine type or number of doses received by each individual. Notably, the observed vaccination rates (peak ~6%) are markedly lower than the contemporaneous national coverage (57%), suggesting under-capture of vaccine records in the database [61]. Therefore, while temporal proximity was observed, the true impact of vaccination on HZ risk could not be determined. This limitation highlights the need for future studies incorporating complete immunization data to clarify the potential role of vaccination timing and type in HZ pathogenesis.

Beyond vitamin D-related mechanisms, emerging global data suggest that COVID-19 may have lasting effects on immune regulation, potentially increasing the susceptibility to autoimmune or inflammatory conditions. Multinational cohort studies from South Korea, Japan, and the UK have reported a rise in post-COVID allergic and immune-related diseases, particularly in children, suggesting a broader shift in immune homeostasis [8]. Additionally, pandemic-related changes in healthcare-seeking behaviors—such as diagnostic delays and rebound clinic visits—may influence the timing and recognition of autoimmune conditions in real-world data [62,63]. These factors provide relevant context for interpreting our findings and highlight the need for future mechanistic studies.

To address potential surveillance bias, we analyzed baseline healthcare utilization using ICD-10 Z-codes (e.g., general checkups, follow-ups). Before matching, the HZ group showed higher Z-code frequencies (SMDs up to 0.42), but after PSM, all SMDs dropped below 0.01, indicating adequate balance. Sensitivity analyses with Z-codes included in the matching model (Table S4) showed attenuated but still significant HRs across all outcomes, suggesting that increased detection alone does not fully explain the observed associations. Furthermore, although unmeasured confounding cannot be ruled out, the consistency of results after adjusting for a wide range of demographic, clinical, and laboratory covariates strengthens the inference of a true association between post-COVID HZ and adverse outcomes.

Several methodological and data-related limitations should be acknowledged when interpreting the results of this study. First, although rigorous 1:1 PSM was applied, some laboratory variables such as ferritin and lactate remained imbalanced post-matching, suggesting possible residual confounding related to systemic inflammation or metabolic stress. Second, the observational nature of this retrospective analysis precludes any causal inference regarding the association between HZ and subsequent renal, infectious, or autoimmune outcomes. Third, exposures and outcomes were identified using ICD-10-CM codes without external validation through medication data, laboratory confirmation, or encounter type, which may introduce misclassification bias. For example, the composite definition of MAKE combines heterogeneous conditions—from AKI to chronic dialysis—which may differ in pathophysiology. Similarly, the use of a single ICD code (A41.9) for sepsis may inadequately capture the full clinical spectrum and could result in under-detection. Fourth, although efforts were made to mitigate detection bias, including matching on baseline healthcare utilization using Z-codes and conducting sensitivity analyses with additional adjustment for healthcare visits, residual differences in surveillance intensity cannot be entirely excluded. Fifth, several important clinical factors—such as COVID-19 severity (e.g., hospitalization or ICU admission), receipt of antivirals or immunosuppressive agents, and COVID-19 or HZ vaccination status—were not available in the dataset and therefore could not be adjusted for. Future studies should incorporate detailed treatment and immunization data to reduce confounding and strengthen causal inference. Sixth, outcome definitions relied solely on the timing of diagnosis codes and did not allow detailed stratification relative to the exact onset of HZ. Seventh, although we analyzed the timing of COVID-19 vaccination relative to HZ reactivation (Supplementary Figure S1 and Table S5), granular information such as vaccine type (e.g., mRNA vs. viral vector), number of doses received, and exact administration dates was not consistently available in the TriNetX platform. In addition, the observed vaccination prevalence (peak ~6%) was markedly lower than national COVID-19 vaccination coverage during the same period (>30%), suggesting likely under-capture of immunization records. These limitations hinder accurate assessment of dose–response relationships or vaccine-specific risks. Eighth, the use of the TriNetX platform imposed constraints on data extraction and matching, resulting in the restriction of the primary analysis to patients aged 50–60 years, which may limit generalizability to younger or older adults. Ninth, while the discussion highlights immune dysregulation as a plausible mechanism linking HZ to renal and autoimmune events, no biomarker or immunologic data were available to support mechanistic hypotheses. Tenth, although hazard ratios were statistically significant, some outcomes—such as SLE—occurred at low absolute rates (e.g., 0.5% vs. 0.2%), and absolute risk differences were modest, warranting cautious interpretation of clinical significance. Lastly, the follow-up period was limited to three years, which may be insufficient to capture long-latency events such as chronic autoimmune or renal conditions manifesting beyond this timeframe. Nevertheless, given the observational nature of this study, all findings should be interpreted as associations rather than causal relationships. Future prospective or mechanistic studies are needed to confirm the temporal and biological links between HZ and these complications.

## 5. Conclusions

This global propensity-matched cohort study demonstrated that HZ diagnosed following COVID-19 is significantly associated with increased risks of MAKE, sepsis, SLE, and RA. These associations remained robust across multiple analytic approaches and sensitivity analyses, independent of baseline comorbidities and laboratory parameters. The findings suggest that HZ following COVID-19 may serve as a clinical indicator of underlying immune dysregulation and heightened vulnerability to renal, infectious, and autoimmune complications. Given the growing burden of post-COVID conditions, early recognition of HZ episodes and proactive surveillance for downstream sequelae may offer an opportunity for timely intervention in high-risk populations.

## Figures and Tables

**Table 1:** Baseline characteristics of COVID-19 patients aged 50–60 years before and after propensity score matching.

	Before Matching				After Matching ^b^			
Characteristics ^a^	COVID-19 with HZ (*n* = 5209)	COVID-19 Without HZ (*n* = 1,848,644)	*p* Value	SMD	COVID-19 with HZ (*n* = 5208)	COVID-19 Without HZ (*n* = 5208)	*p* Value	SMD
Demographics								
Age at Index, mean ± SD	51.2 ± 3.2	50.9 ± 3.2	<0.01 *	0.09	51.2 ± 3.2	51.2 ± 3.2	0.60	0.01 *
Female (%)	63.1%	53.9%	<0.01 *	0.19	63.1%	62.3%	0.38	0.02
Male (%)	33.8%	44.4%	<0.01 *	0.22	33.8%	34.7%	0.38	0.02
White (%)	67.2%	55.3%	<0.01 *	0.25	67.3%	69.1%	0.05 *	0.04
Black or African American (%)	10.4%	14.2%	<0.01 *	0.12	10.4%	10.3%	0.87	0.01
Asian (%)	5.4%	4.2%	<0.01 *	0.06	5.4%	5.0%	0.29	0.02
Diagnosis (%)								
Diabetes mellitus	12.8%	6.5%	<0.01 *	0.22	12.7%	12.4%	0.55	0.01
Hypertensive diseases	24.1%	12.9%	<0.01 *	0.29	24.1%	23.6%	0.55	0.01
Cerebrovascular diseases	2.1%	1.0%	<0.01 *	0.09	2.1%	2.0%	0.68	0.01
Medication (%)								
Blood glucose regulation agents	11.6%	6.0%	<0.01 *	0.20	11.6%	11.3%	0.58	0.01
Beta blockers	9.3%	4.9%	<0.01 *	0.17	9.3%	8.9%	0.50	0.01
Antilipidemic agents	11.9%	6.8%	<0.01 *	0.18	11.9%	12.3%	0.49	0.01
Angiotensin II inhibitor	5.0%	3.0%	<0.01 *	0.10	5.0%	4.6%	0.39	0.02
Diuretics	9.6%	5.4%	<0.01 *	0.16	9.6%	9.5%	0.84	0.01
Laboratory (mean ± SD)								
Sodium, mmol/L	138.8 ± 3.0	138.9 ± 3.0	0.74	0.01	138.8 ± 3.0	138.8 ± 3.1	0.41	0.03
Potassium, mmol/L	4.1 ± 0.5	4.1 ± 0.5	0.44	0.02	4.1 ± 0.5	4.2 ± 0.5	0.20	0.04
Hemoglobin, g/dL	13.2 ± 2.0	13.5 ± 2.0	<0.01 *	0.13	13.2 ± 2.0	13.4 ±1.9	0.01 *	0.09
Hematocrit, %	39.8 ± 6.7	39.7 ± 8.6	0.37	0.02	39.8 ± 6.7	40.0 ± 7.2	0.52	0.02
Leukocytes, 10^3^/uL	60.9 ± 462.2	20.2 ± 229.9	<0.01 *	0.11	58.9 ± 454.4	25.8 ± 274.4	0.01 *	0.09
Platelets, 10^3^/uL	257.2 ± 87.5	260.5 ± 84.5	0.08	0.04	257.2 ± 87.5	258.0 ± 83.4	0.75	0.01
Lymphocytes/100 WBC, %	27.2 ± 11.4	27.1 ± 10.9	0.81	0.01	27.2 ± 11.4	27.3 ± 10.9	0.75	0.01
Iron, ug/dL	71.0 ± 43.2	73.4 ± 44.7	0.41	0.06	71.0 ± 43.2	71.4 ± 40.9	0.93	0.01
Ferritin, ng/mL	317.4 ± 682.6	271.4 ± 868.7	0.40	0.06	317.0 ± 684.0	235.0 ± 482.5	0.13	0.14
Creatinine, mg/dL	1.1 ± 1.3	1.0 ± 1.5	0.16	0.03	1.1 ± 1.3	1.0 ± 1.0	0.01 *	0.08
BUN, mg/dL	16.7 ± 11.4	15.7 ± 9.4	<0.01 *	0.10	16.7 ± 11.4	15.9 ± 8.6	0.01 *	0.09
Bicarbonate, mmol/L	25.7 ± 3.1	25.8 ± 3.2	0.19	0.03	25.7 ± 3.1	25.8 ± 3.3	0.33	0.03
Glucose, mg/dL	118.2 ± 58.2	116.9 ± 55.2	0.26	0.02	118.2 ± 58.2	120.8 ± 63.6	0.17	0.04
ALT, U/L	28.2 ± 40.2	31.3 ± 69.9	0.05 *	0.06	28.1 ± 39.9	31.7 ± 51.3	0.01 *	0.08
AST, U/L	27.2 ± 28.4	30.4 ± 96.0	0.14	0.05	27.1± 28.2	29.4 ± 37.1	0.03 *	0.07
Alk phosphatase, U/L	89.3 ± 45.6	88.1 ± 61.0	0.41	0.02	89.3 ± 45.6	89.0 ± 71.4	0.88	0.01
Albumin, g/dL	4.1 ± 0.5	4.1 ± 0.5	0.33	0.02	4.1 ± 0.5	4.1 ± 0.5	0.54	0.02
Protein, g/dL	7.1 ± 0.7	7.2 ± 0.8	0.01 *	0.09	7.1 ± 0.7	7.1 ± 0.7	0.20	0.04
Cholesterol, mg/dL	192.2 ± 43.9	190.4 ± 44.7	0.19	0.04	192.2 ± 43.9	189.3 ± 44.6	0.13	0.065
LDL Cholesterol, mg/dL	111.2 ± 36.3	111.0 ± 37.3	0.81	0.01	111.2 ± 36.3	109.7 ± 38.3	0.34	0.04
HDL Cholesterol, mg/dL	49.1 ± 20.7	48.5 ± 20.1	0.37	0.03	49.1 ± 20.7	48.1 ± 20.5	0.27	0.05
Triglyceride, mg/dL	151.8 ± 128.5	150.2 ± 145.5	0.72	0.01	151.8 ± 128.5	155.6 ± 153.0	0.53	0.03
HbA1c, %	6.7 ± 2.0	6.6 ±1.9	0.13	0.05	6.7 ± 2.0	6.7 ± 2.1	0.46	0.03
Calcidiol, ng/mL	34.2 ± 16.8	32.7 ± 16.7	0.15	0.09	34.2 ± 16.8	34.7 ± 18.1	0.79	0.03
CRP, mg/L	17.7 ± 49.4	24.6 ± 51.3	0.02 *	0.14	17.7 ± 49.4	19.2 ± 40.2	0.69	0.04
ESR, mm/h	22.8 ± 23.9	25.0 ± 25.5	0.17	0.09	22.8 ± 23.9	22.4 ± 22.2	0.87	0.02
Lactate, mmol/L	1.4 ± 0.7	1.6 ± 1.5	0.32	0.12	1.4 ± 0.7	1.5± 0.7	0.40	0.12
Urate, mg/dL	5.6 ± 1.8	5.8 ± 2.1	0.52	0.06	5.6 ± 1.8	5.6 ± 1.8	0.98	0.01

Abbreviations: ALT, alanine aminotransferase; AST, aspartate aminotransferase; BUN, blood urea nitrogen; CRP, C-reactive protein; ESR, erythrocyte sedimentation rate; HbA1c, hemoglobin A1c; HDL, high-density lipoprotein; HZ, herpes zoster; LDL, low-density lipoprotein; SD, standard deviation; SMD, standardized mean difference. ^a^. Data are presented as % unless otherwise indicated. ^b^. Propensity score matching was performed using age at index, sex, and race. * *p* < 0.05, indicating statistical significance.

## Data Availability

Due to licensing and privacy restrictions, the data used in this study from the TriNetX Global Health Research Network are not publicly available. TriNetX provides access to de-identified, aggregate-level data obtained from a global network of healthcare organizations. Researchers may request access through the TriNetX website (https://trinetx.com/) or by contacting Privacy@TriNetX.com. Data are available on reasonable request from the corresponding author.

## Supplementary Materials

The following supporting information is available at https://www.mdpi.com/article/10.3390/biomedicines13071628/s1. Table S1: Three-year outcome risks based on Kaplan–Meier analysis of the full cohort and age-restricted subgroup (50–60 years). Table S2: Three-year clinical outcome risks derived from Kaplan–Meier survival curves before and after propensity score matching. Table S3. Assessment of surveillance bias using ICD-10 Z-Code frequencies before and after matching. Table S4. Comparison of three-year outcome risks before and after including healthcare utilization (Z-Code) covariates in propensity score matching. Table S5. Timing and prevalence of COVID-19 vaccination relative to HZ reactivation. Figure S1. Vaccination prevalence and timing in the HZ cohort.

## Abbreviations

AKI	Acute Kidney Injury
ALT	Alanine Aminotransferase
AST	Aspartate Aminotransferase
BUN	Blood Urea Nitrogen
BMI	Body Mass Index
CKD	Chronic Kidney Disease
CKD-EPI	Chronic Kidney Disease Epidemiology Collaboration
CMV	Cytomegalovirus
COPD	Chronic Obstructive Pulmonary Disease
COVID-19	Coronavirus Disease 2019
CRP	C-Reactive Protein
CPT	Current Procedural Terminology
EBV	Epstein–Barr Virus
eGFR	Estimated Glomerular Filtration Rate
ESR	Erythrocyte Sedimentation Rate
HbA1c	Hemoglobin A1c
HDL	High-Density Lipoprotein
HSV	Herpes Simplex Virus
HZ	Herpes Zoster
IL-6	Interleukin-6
LDL	Low-Density Lipoprotein
MAKE	Major Adverse Kidney Events
PSM	Propensity Score Matching
RA	Rheumatoid Arthritis
SARS-CoV-2	Severe Acute Respiratory Syndrome Coronavirus 2
SLE	Systemic Lupus Erythematosus
TNF-α	Tumor Necrosis Factor Alpha
VZV	Varicella-Zoster Virus
