# Supplementary material for: Herpes Zoster Reactivation Following COVID-19 and the Risk of Renal, Infectious, and Autoimmune Complications: A Global Propensity-Matched Cohort Study
Source: Biomedicines. 2025 Jul 2;13(7):1628. doi: 10.3390/biomedicines13071628 (PMC12292594; doi:10.3390/biomedicines13071628)

Supplementary Table S1. Three-year outcome risks based on Kaplan–Meier analysis of the full cohort and age-restricted subgroup (50–60 years).

Outcomes	PSM	HR[a]	95% CI (Lower)[a]	95% CI (Upper)[a]	p value Log-Rank Test
**MAKE**	Entire age	1.940	1.866	2.017	< 0.0001
	Age 50~60	1.473	1.324	1.638	< 0.0001
**Sepsis**	Entire age	2.362	2.250	2.479	< 0.0001
	Age 50~60	2.063	1.181	2.352	< 0.0001
**SLE**	Entire age	2.667	2.254	3.156	< 0.0001
	Age 50~60	1.877	1.234	2.856	< 0.005
**RA**	Entire age	2.488	2.267	2.730	< 0.0001
	Age 50~60	2.468	2.019	3.017	< 0.0001

Abbreviations: CI: Confidence Interval; HR: Hazard Ratio; MAKE: Major Adverse Kidney Events; PSM: Propensity Score Matching; RA: Rheumatoid Arthritis; SLE: Systemic Lupus Erythematosus.

a. Hazard ratio was adjusted using age at index, sex, race.

Supplementary Table S2. Three-year clinical outcome risks derived from Kaplan–Meier survival curves before and after propensity score matching.

Outcomes	PSM	HR[a]	95% CI (Lower)[a]	95% CI (Upper)[a]	p value Log-Rank Test
**MAKE**	Before	1.934	1.861	2.011	< 0.0001
	After	1.940	1.866	2.017	< 0.0001
**Sepsis**	Before	2.352	2.239	2.470	< 0.0001
	After	2.362	2.250	2.479	< 0.0001
**SLE**	Before	2.615	2.205	3.102	< 0.0001
	After	2.667	2.254	3.156	< 0.0001
**RA**	Before	2.484	2.262	2.727	< 0.0001
	After	2.488	2.267	2.730	< 0.0001

Abbreviations: CI: Confidence Interval; HR: Hazard Ratio; MAKE: Major Adverse Kidney Events; PSM: Propensity Score Matching; RA: Rheumatoid Arthritis; SLE: Systemic Lupus Erythematosus.

a. Hazard ratio was adjusted using age at index, sex, race.

Supplementary Table S3. Assessment of surveillance bias using ICD-10 Z-Code frequencies before and after matching.

	Before Matching				After Matching			
**Characteristics**	**COVID-19 with HZ (n=5,209)**	**COVID-19 without HZ (n=1,848,644)**	**p value**	**Std diff**	**COVID-19 with HZ (n=5,208)**	**COVID-19 without HZ (n=5,208)**	**p value**	**Std diff**
**Diagnosis (%)**								
**Factors influencing health status (Z00–Z99)**	51.8%	31.6%	<0.01	0.42	51.8%	52.0%	0.86	< 0.01
**Persons encountering health services for examinations (Z00–Z01)**	33.4%	19.7%	<0.01	0.31	33.4%	33.8%	0.67	< 0.01
**Persons with potential health hazards related to family and personal history (Z80–Z99)**	29.1%	15.1%	<0.01	0.34	29.1%	29.2%	0.92	< 0.01
**Persons with potential health hazards related to communicable diseases (Z20–Z29)**	19.2%	10.7%	<0.01	0.24	19.2%	19.2%	0.96	< 0.01
**Encounters for other specific health care (Z40–Z53)**	7.0%	2.8%	<0.01	0.19	7.0%	7.1%	0.94	< 0.01

**Persons encountering health services in other circumstances (Z60–Z76)**	5.9%	3.1%	<0.01	0.14	5.9%	6.1%	0.72	< 0.01

Supplementary Table S4. Comparison of three-year outcome risks before and after including healthcare utilization (Z-Code) covariates in propensity score matching.

Outcomes	PSM	HR[a]	95% CI (Lower)[a]	95% CI (Upper)[a]	p value Log-Rank Test
**MAKE**	Original cohort	1.940	1.866	2.017	0.001
	With Z-code adjustment	1.587	1.331	1.891	< 0.0001
**Sepsis**	Original cohort	2.362	2.250	2.479	< 0.0001
	With Z-code adjustment	1.620	1.306	2.010	< 0.0001
**SLE**	Original cohort	2.667	2.254	3.156	< 0.0001
	With Z-code adjustment	2.505	1.174	5.345	0.014
**RA**	Original cohort	2.488	2.267	2.730	< 0.0001
	With Z-code adjustment	2.111	1.460	3.051	< 0.0001

Abbreviations: CI: Confidence Interval; HR: Hazard Ratio; MAKE: Major Adverse Kidney Events; PSM: Propensity Score Matching; RA: Rheumatoid Arthritis; SLE: Systemic Lupus Erythematosus.

a. Hazard ratio was adjusted using age at index, sex, race.

Supplementary Table S5. Timing and prevalence of COVID-19 vaccination relative to HZ reactivation.

**Time Interval Relative to HZ Reactivation**	**Number vaccinated for COVID-19**	**% Vaccinated for COVID-19**
15-18 months before	400	1.35%
12-15 months before	613	2.07%
9-12 months before	837	2.83%
6-9 months before	1165	3.93%
3-6 months before	1438	4.86%
0-3 months before	1717	5.80%
0-3 months after	1765	5.96%
3-6 months after	1620	5.47%
6-9 months after	1316	4.44%
9-12 months after	1051	3.55%
12-15 months after	814	2.75%
15-18 months after	684	2.31%

Supplementary Figure S1. Vaccination prevalence and timing in the HZ cohort.

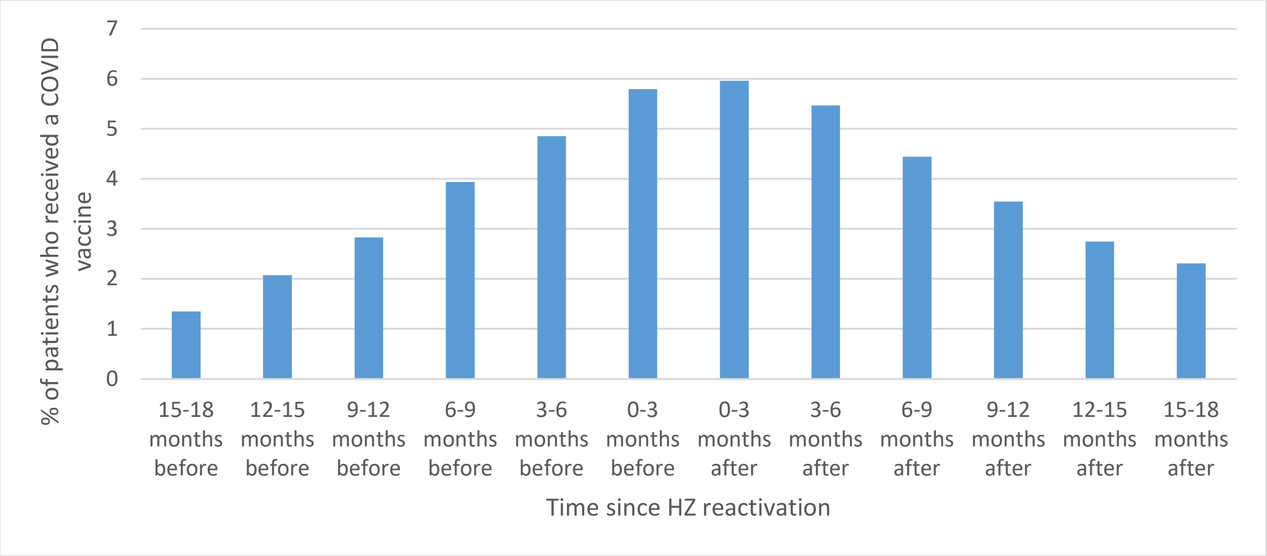

Supplement: Supplementary file 1 [file biomedicines-13-01628-s001.zip › biomedicines-3688636-supplementary.pdf]
